# Supplementary material for: The therapeutic potential of multiclonal tumoricidal T cells derived from tumor infiltrating lymphocyte-derived iPS cells
Source: Commun Biol. 2021 Jun 7;4:694. doi: 10.1038/s42003-021-02195-x (PMC8184746; doi:10.1038/s42003-021-02195-x)
Supplement: Supplementary file 2 — Supplementary Information [file 42003_2021_2195_MOESM2_ESM.pdf]

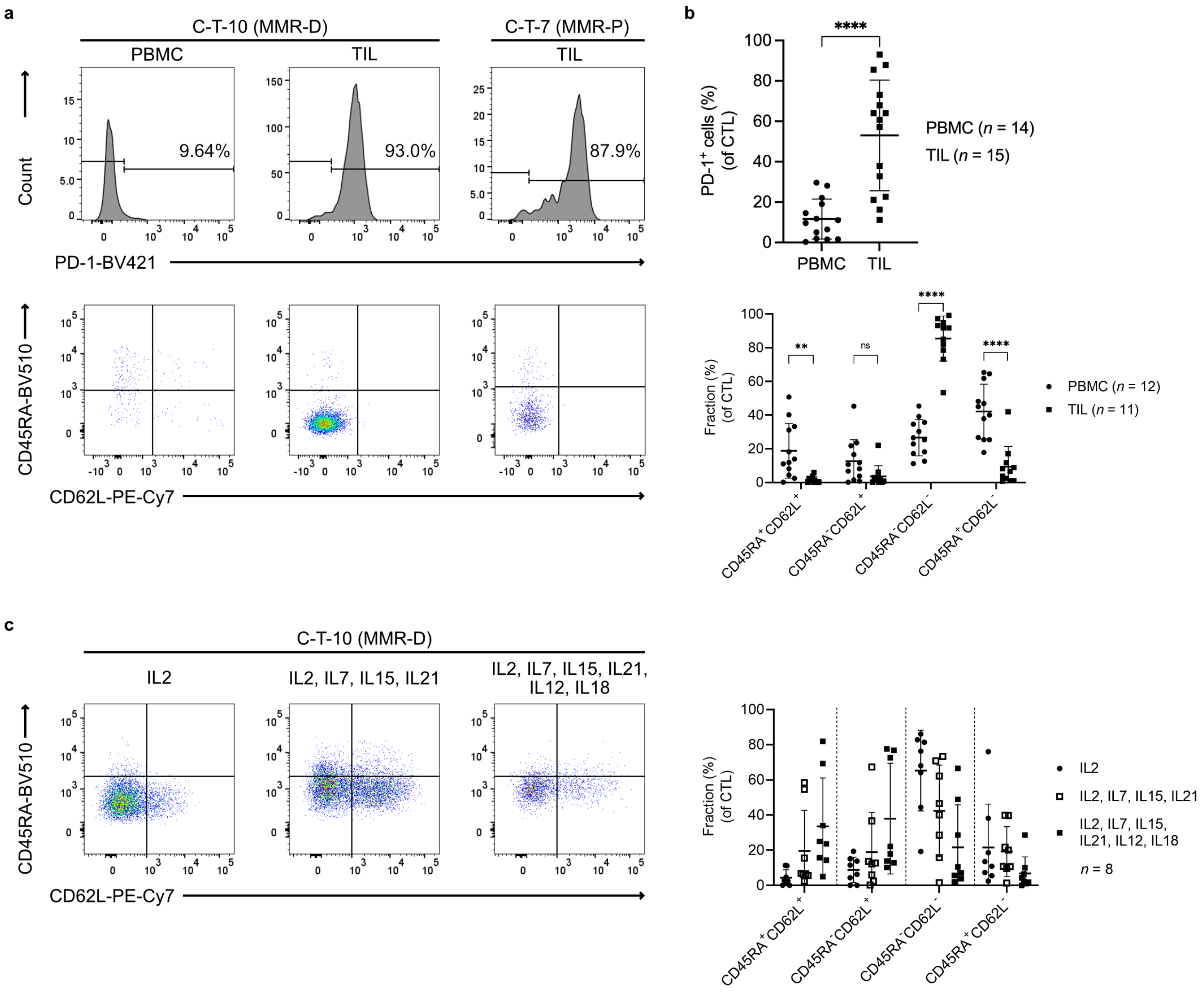

Supplementary Fig. 1 | Profiles of pre- and post-cytokine expansion TIL from human colorectal cancer. a, b Profiles of PBMC and pre-expansion TIL. **a** Representative flow cytometry data. Data were gated on $CD3^{+}CD4^{-}CD8^{+}$ live cells. **b** Percentages of different cell populations. Dots represent data from individual cases. Means are shown; error bars represent SD. Top: $^{****}P <$ 0.0001, two-tailed unpaired t test. Bottom: $^{**}P < 0.01$; $^{****}P < 0.0001$, NS, not significant, two-way ANOVA followed by Sidak's multiple comparisons test. **c** Phenotypes of post-cytokine expansion TIL. Left: representative flow cytometry plots from each cytokine condition. Data were gated on $CD3^{+}CD4^{-}CD8^{+}$ live cells. Right: Phenotype fractions of post-cytokine expansion TIL are indicated. Dots represent data from individual cases. Means are shown; error bars represent SD.

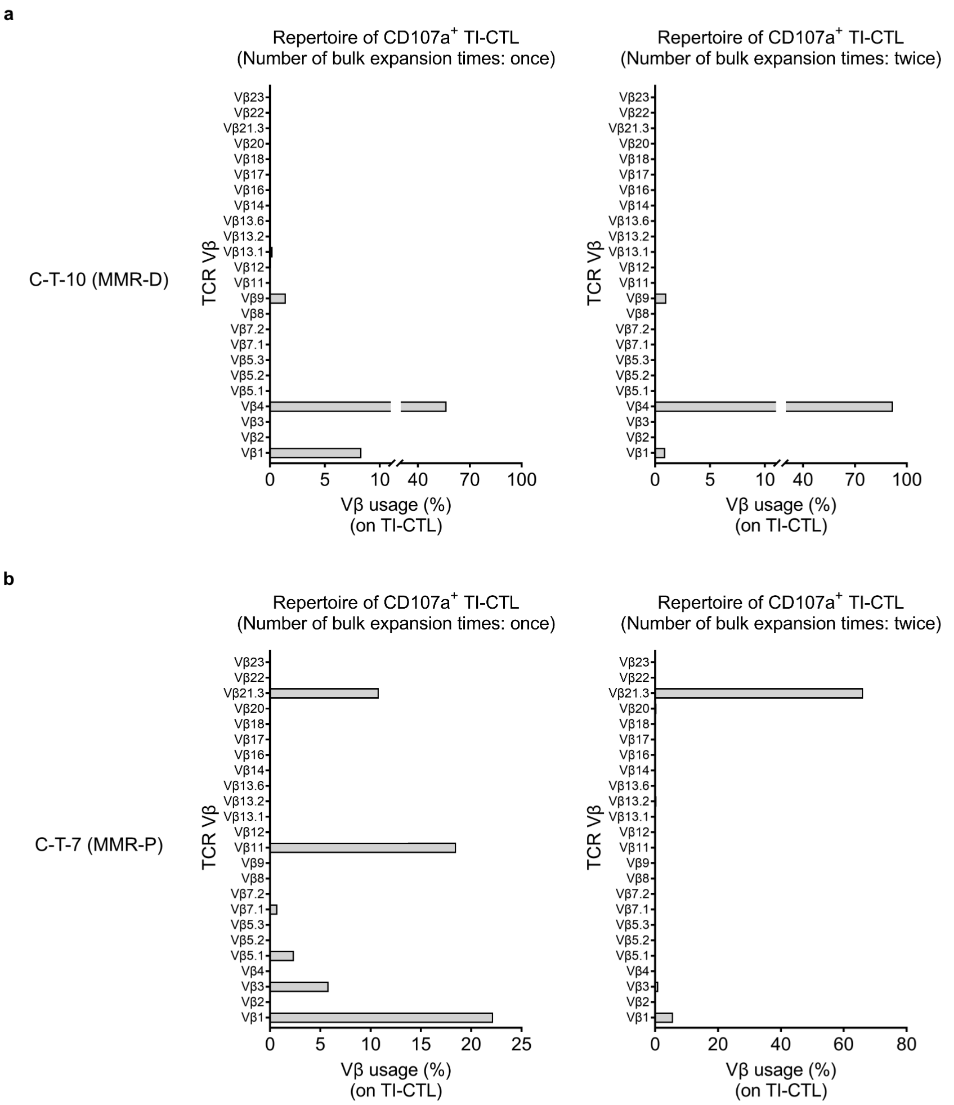

Supplementary Fig. 2 | Repetitive bulk expansion of tumor-reactive TI-CTL leads to a narrower diversity. a, b TCR Vβ repertoire analysis of the tumor-reactive TI-CTL after co-culturing with cancer spheroids and expanded in the bulk state. Data from C-T-10 MMR-D (**a**) and C-T-7 MMR-P (**b**). Left: the number of bulk expansions was one. Right: the number of bulk expansions was two.

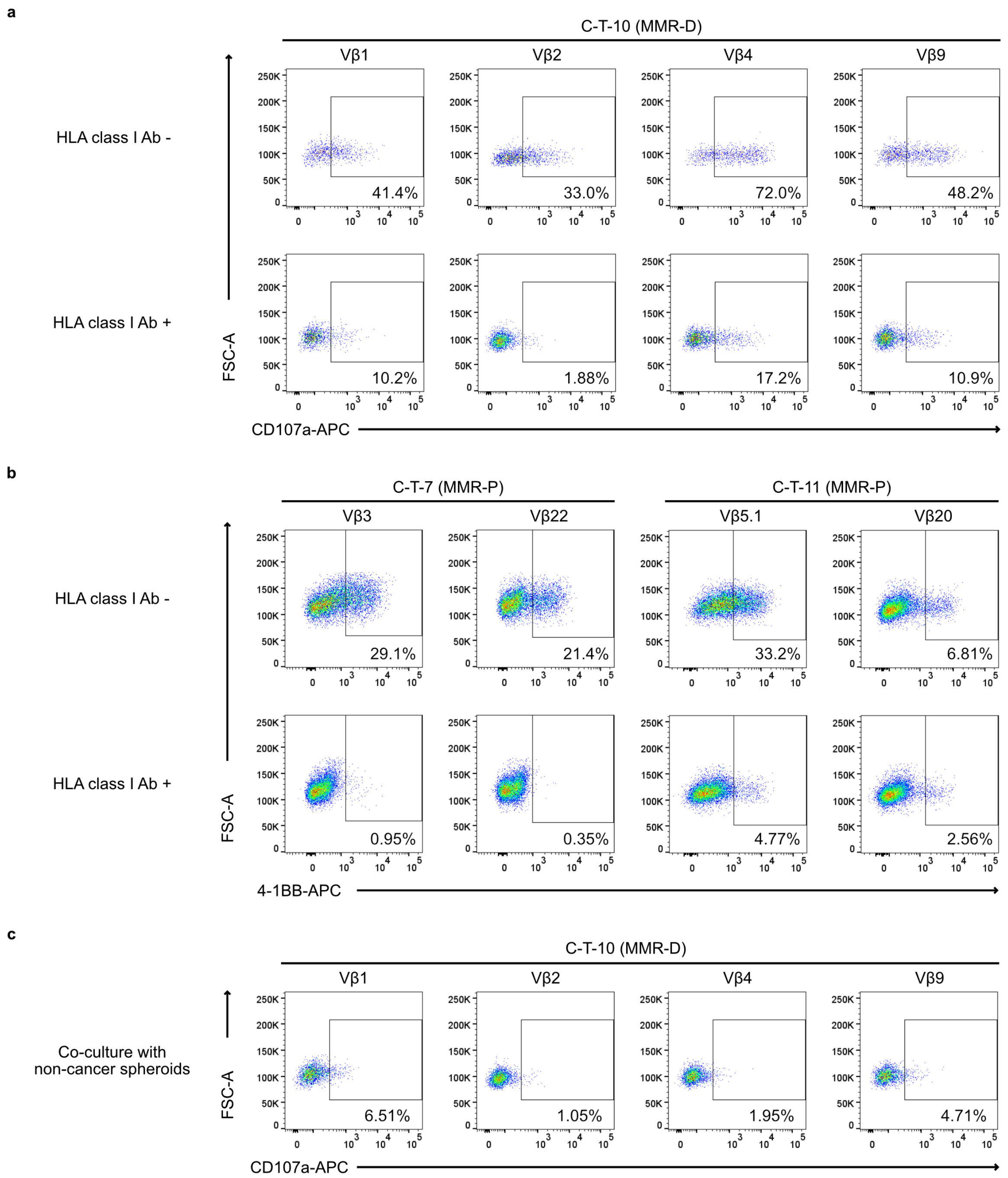

Supplementary Fig. 3 I Vβ-based TI-CTL reactivity against autologous spheroids. a, b Flow cytometry plots of Vβ-based TI-CTL reactivity against autologous cancer spheroids with or without HLA class I blocking antibody. Data from MMR-D (**a**) and MMR-P (**b**). Representative data of 2 independent experiments for C-T-7 and 3 independent experiments for C-T-10 and C-T-11. All data were gated on CD45$^+$ live cells. **c** Flow cytometry data from C-T-10 MMR-D for reactivity against autologous non-cancer spheroids. Representative data of 3 independent experiments. Data were gated on CD45$^+$ live cells.

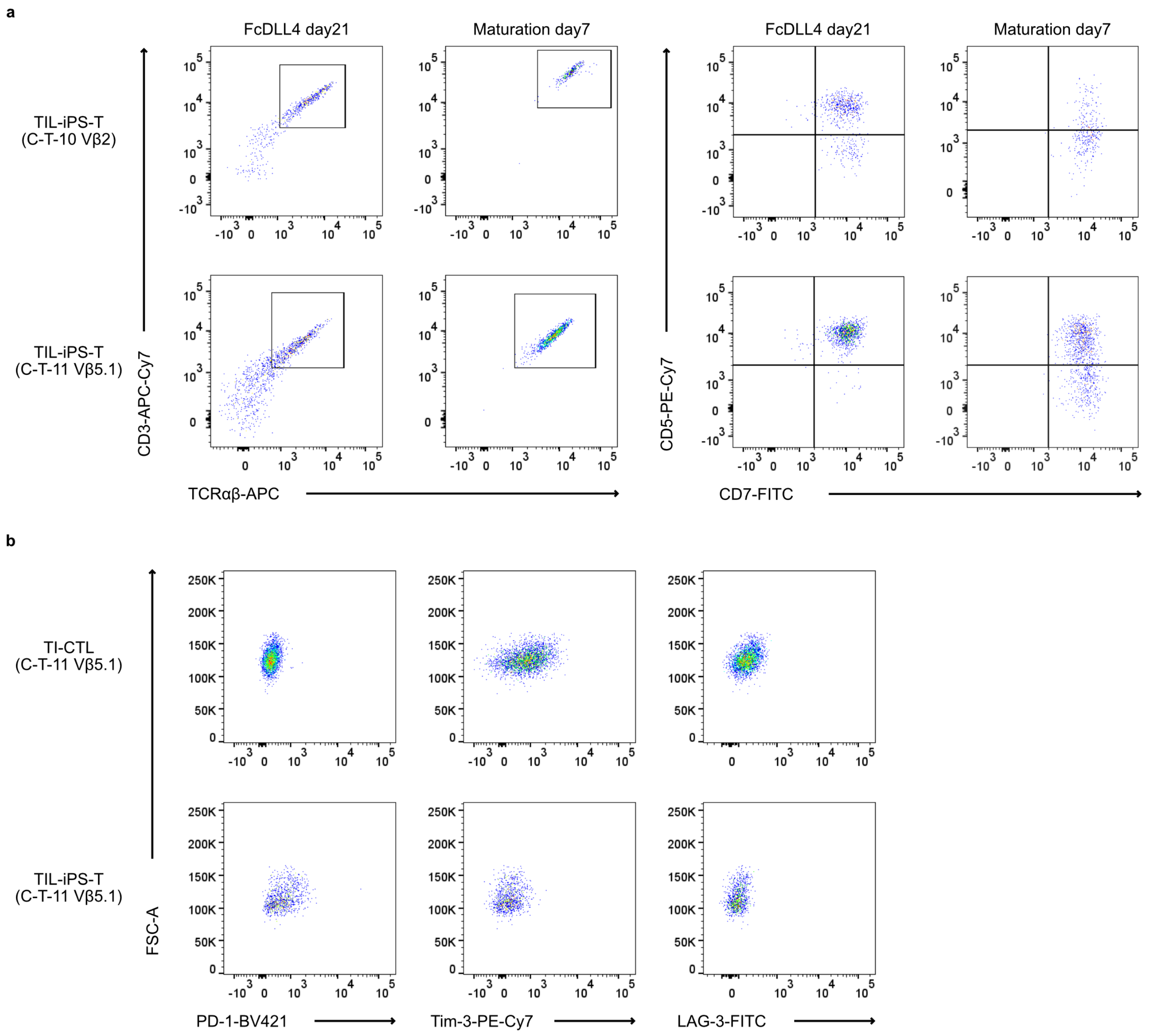

Supplementary Fig. 4 I Surface marker profiles of TIL-iPS-T. a Flow cytometry analysis of T cell lineage markers at each differentiation stage. All data were gated on $CD45^+CD4^-CD8\alpha^+CD8\beta^+$ live cells. **b** Flow cytometry analysis of exhaustion-related markers on TI-CTL and TIL-iPS-T. All data were acquired from C-T-11 Vβ5.1 clone. The data for TIL-iPS-T were acquired 21 days after culturing on FcDLL4. All data were gated on $CD3^+CD4^-CD8\alpha^+CD8\beta^+$ live cells.

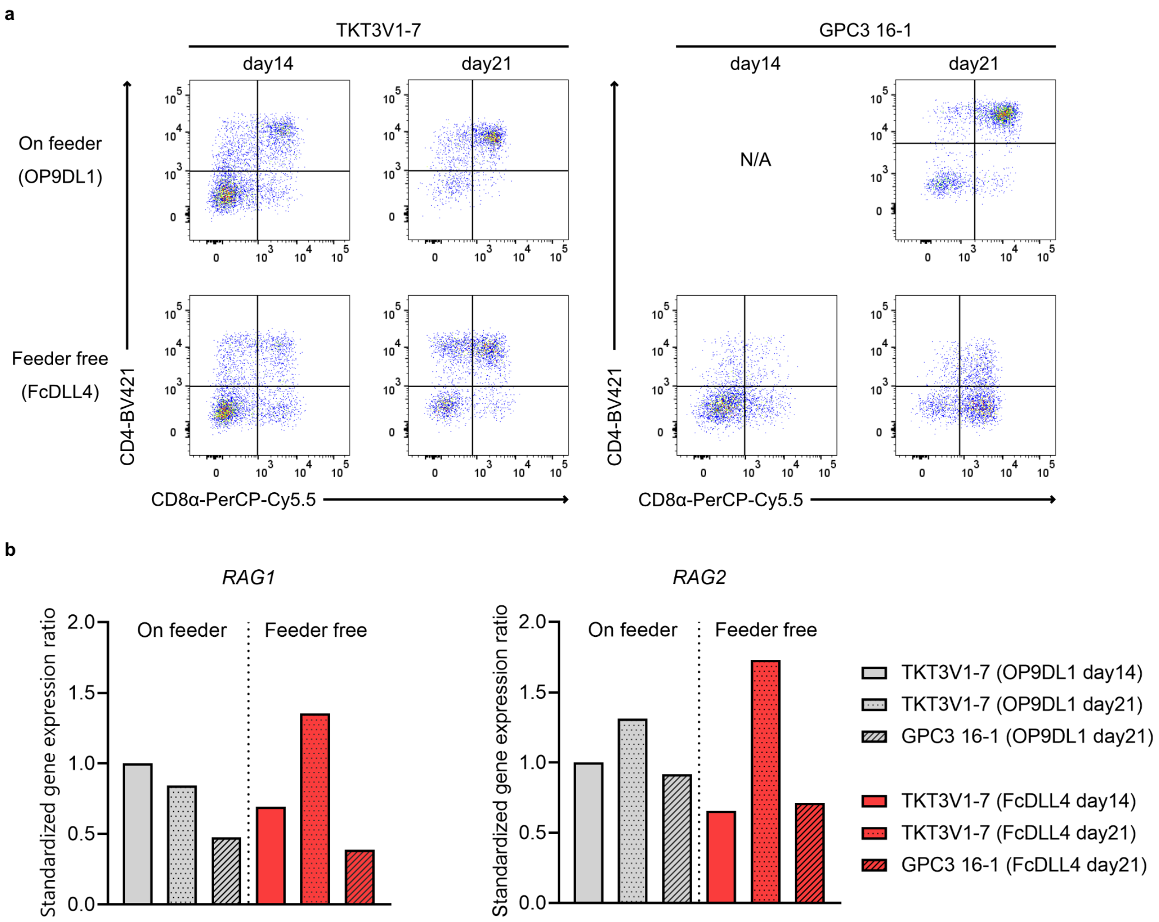

Supplementary Fig. 5 l Comparison of *RAG1* and *RAG2* expression on T cell-derived iPS-T between OF and FF differentiation protocols. a Flow cytometry analysis indicating T cell differentiation from T-iPSC clones. Data were acquired on days 14 and 21 for both differentiation protocols. DP populations were sorted for the following experiment. Data were gated on $CD45^+$ live cells. **b** *RAG1* and *RAG2* expressions on DP cells were quantified by qPCR. Representative data of 2 independent experiments. The expression levels were standardized with the ΔCt method by *GAPDH* expression and further standardized with the ΔΔCt method by the ΔCt mean of *RAG1* or *RAG2* expression in DP cells from TKT3V1-7 14 days after culturing on OP9DL1.

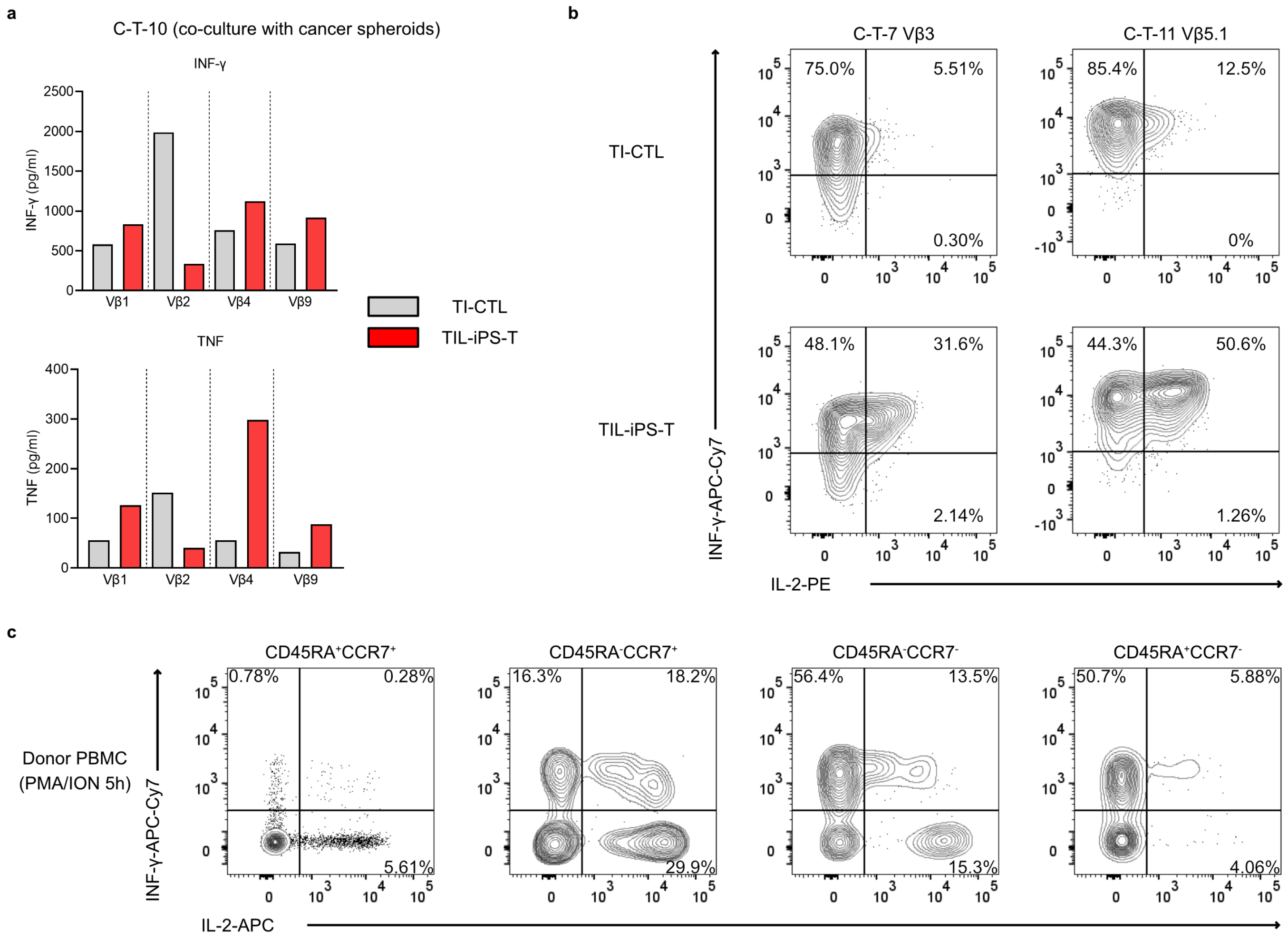

Supplementary Fig. 6 I Cytokine-producing function of TIL-iPS-T. a Cytometric beads array (CBA) data for INF-γ and TNF are indicated. Both TI-CTL and TIL-iPS-T for C-T-10 were stimulated by co-culturing with cancer spheroids. The data were acquired after 48 h co-culture with cancer spheroids. **b** Cytokine production capacity was evaluated for MMR-P by stimulating with PMA plus ION. Representative data of 3 independent experiments are shown. Data were gated on $CD45^{+}CD4^{-}CD8\alpha^{+}CD8\beta^{+}$ cells. **c** The relationship between the T cell phenotype and cytokine production pattern is indicated. PBMC from healthy donors was used for the experiments. Representative data of 2 independent experiments. Data were gated on $CD3^{+}CD4^{-}CD8^{+}$ cells.

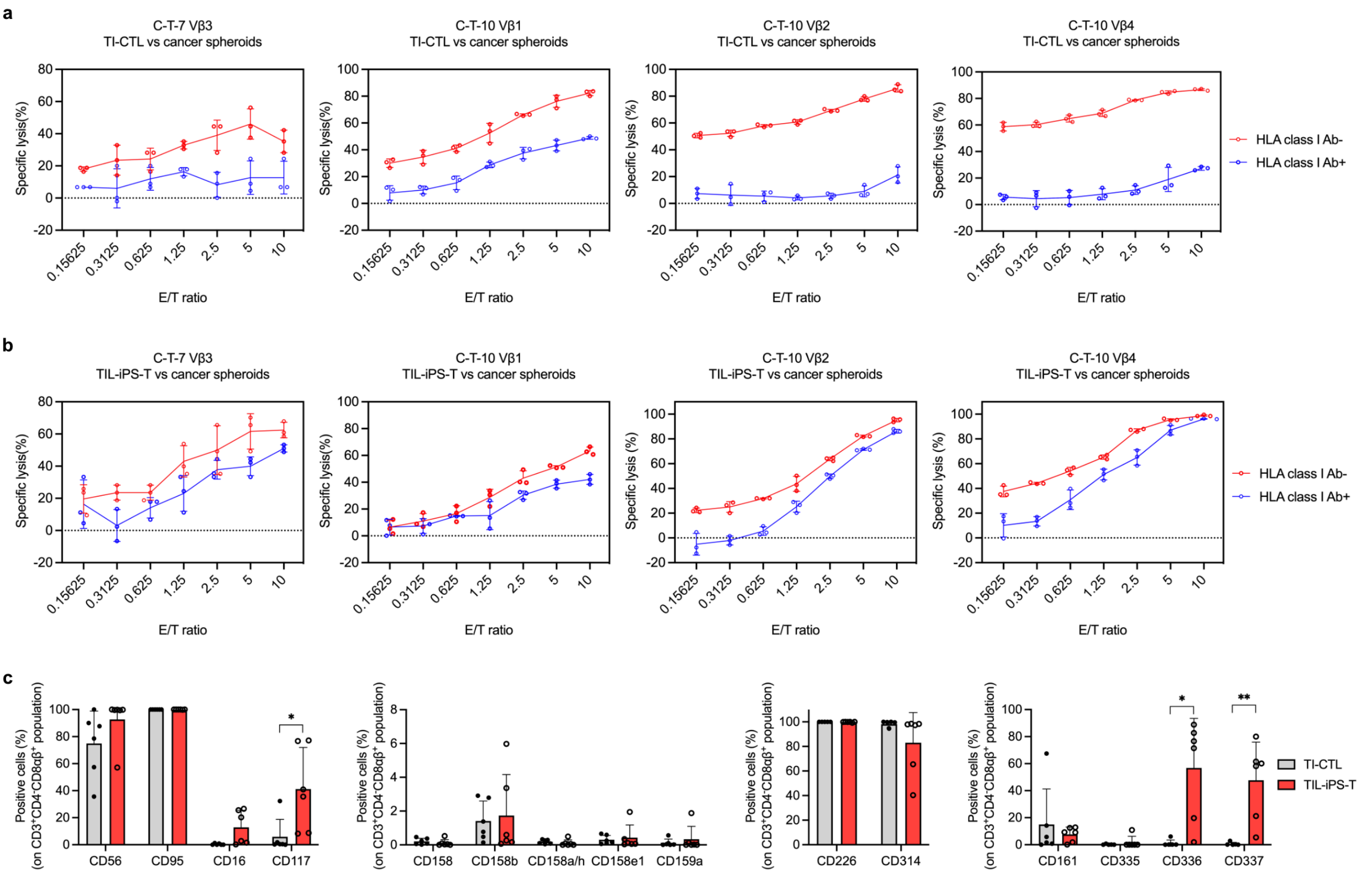

Supplementary Fig. 7 I Additional killing function of TIL-iPS-T independent of TCR-HLA class I interaction. a, b The killing function of tumor-specific TI-CTL clones (**a**) and TIL-iPS-T clones (**b**) was quantified with or without HLA class I blocking antibody. Representative data of 2 independent experiments. Dots represent individual values. Means are connected by lines; error bars represent SD. $n = 3$ per point. **c**, NK cell-related markers on TI-CTL and TIL-iPS-T. Dots represent data from each Vβ clone. Means are indicated by boxes; error bars represent SD. $n = 3$ per point. $^{*}P < 0.05$, $^{**}P < 0.01$, two-tailed paired t test.

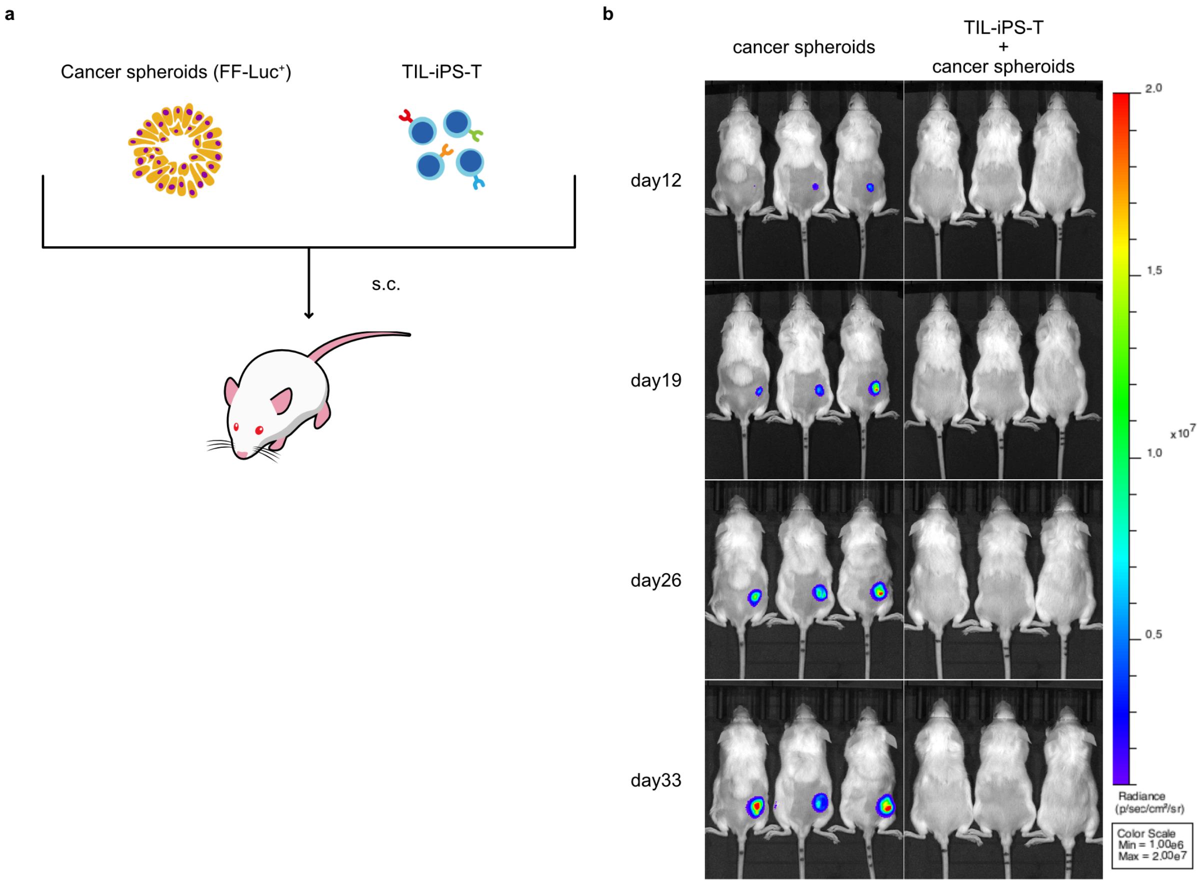

Supplementary Fig. 8 l TIL-iPS-T inhibited engraftment of cancer spheroids in NSG mice. a A schema of the Winn assay. 1 million firefly luciferase-transduced (FF-Luc+) cancer spheroids and TIL-iPS-T each were subcutaneously injected into NSG mice at the same time and place. **b** Bioluminescence imaging of each group by an in vivo imaging system (IVIS). *n* = 3 per point.

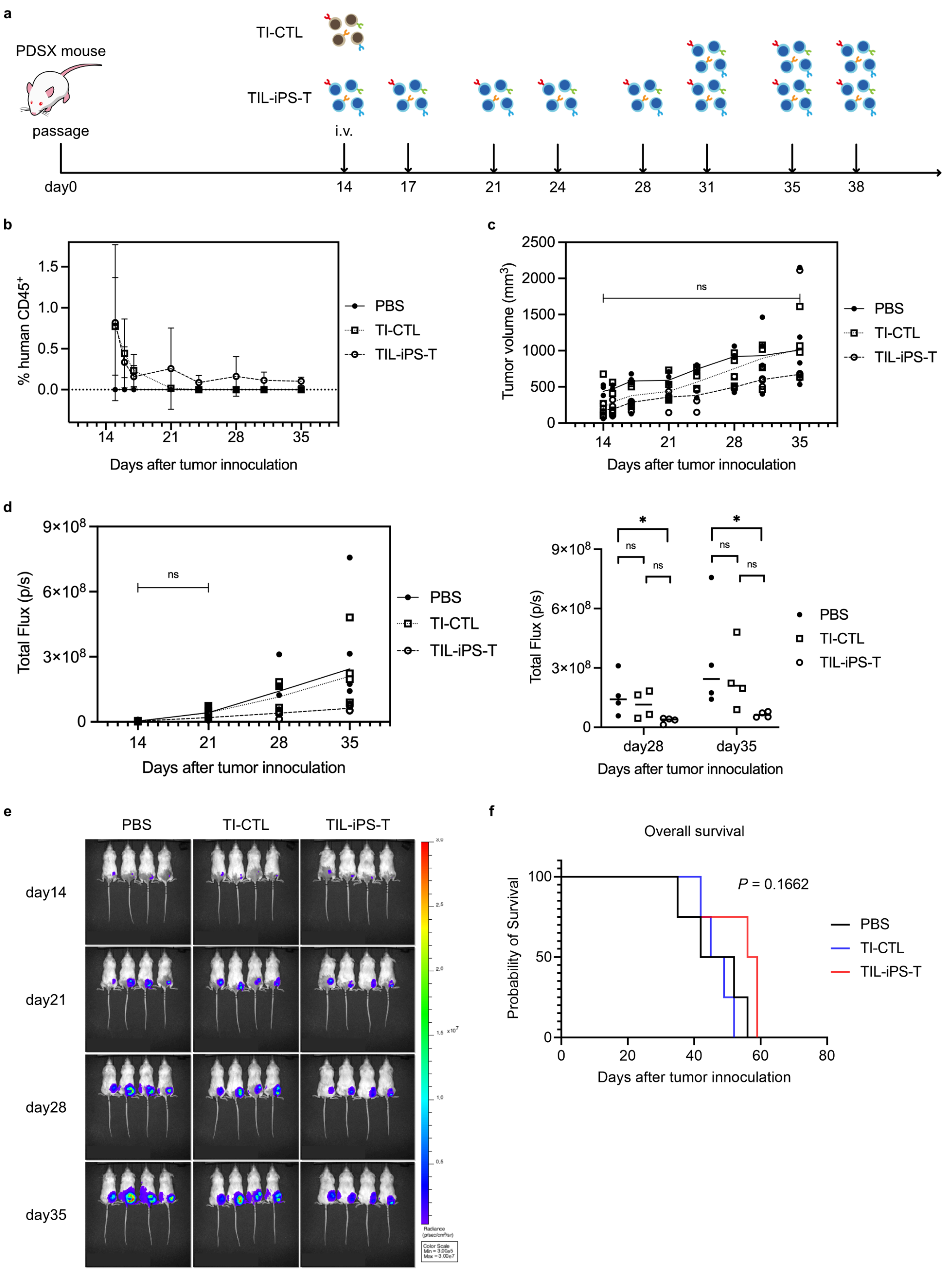

Supplementary Fig. 9 | Evaluating the therapeutic potential of TIL-iPS-T in PDSX mice. a Experimental design for evaluating the therapeutic potential of TIL-iPS-T for C-T-10 MMR-D via intravenous injection. FF-Luc+ cancer spheroids were used to establish PDSX mice bearing subcutaneous tumors. All of the available clones (Vβ1, Vβ2, Vβ4 and Vβ9) were mixed at the same ratio and injected. In the TI-CTL group, 5×10^6 TI-CTL were transfused once. In the TIL-iPS-T group, 5×10^6 TIL-iPS-T were transfused five times followed by three transfusions of 1×10^7 TIL-iPS-T. n = 4 mice per group. **b** The percentage of human CD45+ cells within the mouse PBMC fraction is indicated. Means are shown; error bars represent SD. **c** Tumor volumes in each PDSX mouse were measured at the indicated times. Non-parametric analysis (Kruskal-Wallis test followed by Dunn's multiple comparisons test) was used for the statistical analysis. Dots represent data from individual mice. Medians are shown and connected by lines. NS, not significant. **d** The bioluminescence level of each PDSX mouse was measured at the indicated times. Dots represent data from individual mice. Medians are shown. Left: time course of the bioluminescence level for each group. Medians are connected by lines. Right: statistical comparisons of the bioluminescence levels on days 28 and 35. $^*P < 0.05$, NS, not significant, non-parametric analysis (Kruskal-Wallis test followed by Dunn's multiple comparisons test). **e** Bioluminescence imaging is shown. **f** Kaplan-Meier curve representing the percent survival of each group. The log-rank test was used for the statistical analysis.

Case	Age	Sex	Location of Primary Tumor*	Histology**	DNA mismatch repair profiles of tumor				TNM (UICC 8th)		
					MSH2	MSH6	MLH1	PMS2	T	N	M
C-T-1	78	M	D	tub2					4a	2	0
C-T-2	63	F	A	tub1 > tub2 > muc					3	2	1a
C-T-3	70	M	RS	tub2		intact		intact	4a	1	0
C-T-4	71	M	C	tub2		intact		intact	4a	0	0
C-T-5	74	F	S	tub2 > tub1		intact		intact	3	2	0
C-T-6	80	M	R	tub2	intact	intact	intact	intact	2	0	0
C-T-7	84	M	RS	tub1					1	0	0
C-T-8	75	F	T	tub2		intact		intact	4a	0	0
C-T-9	82	F	S	tub1 > tub2		intact		intact	3	1	0
C-T-10	76	F	C	tub2 > med	intact	intact	loss	loss	3	1	0
C-T-11	72	F	A	tub2		intact		intact	3	0	0
C-T-12	84	M	T	muc > tub2		intact		intact	3	0	0
C-T-13	82	M	S	tub1					2	0	0
C-T-14	63	M	T	tub2		intact		intact	3	0	0
C-T-15	77	M	RS	tub2		intact		intact	is	0	0
			S	tub2 > pap		intact		intact	2	0	0
C-T-16	74	F	S	tub2					3	0	1a

* C: Cecum, A: Ascending colon, T: Transverse colon, D: Descending colon, S: Sigmoid colon, RS: Rectosigmoid junction, R: Rectum

** tub: Tubular adenocarcinoma, tub1: Well differentiated type, tub2: Moderately differentiated type, muc: Mucinous adenocarcinoma, pap: Papillary adenocarcinoma, med: Medullary carcinoma

Supplementary Table 1 | Clinical information of colorectal cancer patients.

Case	Clone	Reprogramming from TI-CTL		T cell regeneration
		Colony number	Colony forming rate (%)	
C-T-7	Vβ3	462	4.62	Success
	Vβ5.3*	TNTC**		Success
	Vβ22	281	2.81	Fail
C-T-10	Vβ1	312	3.12	Success
	Vβ2	145	1.45	Success
	Vβ4	239	2.39	Success
	Vβ9	17	0.17	Success
	Vβ23*	54	0.54	Success
C-T-11	Vβ5.1	59	0.59	Success
	Vβ20	105	1.05	Success

The starting number of TI-CTL was 10,000 cells in all experiments.
* Tumor nonspecific clones
** TNTC: too numerous to count

Supplementary Table 2 | Reprogramming and T cell regeneration profiles of selected TI-CTL.

Dilution	Manufacture	Anti-human antibodies
1:40	BioLegend	CD3-BV510 (clone: UCHT1, 300448), CD3-APC (clone: UCHT1, 300412), CD3-APC-Cy7 (clone: UCHT1, 300426), CD4-BV421 (clone: OKT4, 317434), CD7-FITC (clone: CD7-6B7, 343104), CD8-PerCP-Cy5.5 (clone: SK1, 344710), CD14-PE-Cy7 (clone: HCD14, 325618), CD16-FITC (clone: 3G8, 302006), CD27-APC (clone: O323, 302810), CD28-BV421 (clone: CD28.2, 302930), CD34-PB (clone: 581, 343512), CD45-BV421 (clone: HI30, 304031), CD45-BV510 (clone: HI30, 304036), CD45RA-BV510 (clone: HI100, 304142), CD56-APC-Cy7 (clone: HCD56, 318332), CD62L-PE-Cy7 (clone: DREG-56, 304822), CD95-PE-Cy7 (clone: DX2, 305622), CD107a-PE (clone: H4A3, 320608), 4-1BB (CD137)-PE (clone: 4B4-1, 309804), 4-1BB (CD137)-APC (clone: 4B4-1, 309810), CD158-APC (clone: HP-MA4, 339509), CD158a/h-FITC (clone: HP-MA4, 339503), CD158b-APC (clone: DX27, 312715), CD158e1-APC (clone: DX9, 312715), CD161-PE-Cy7 (clone: HP-3G10, 339918), CCR7 (CD197)-APC (clone: G043H7, 353214), LAG-3 (CD223)-FITC (clone: 11C3C65, 369308), CD226 (DNAM-1)-BV421 (clone: 11A8, 338332), PD-1 (CD279)-BV421 (clone: EH12.2H7, 329920), CD314 (NKG2D)-PE-Cy7 (clone: 1D11, 320811), CD335-FITC (clone: 29A1.4, 331921), CD336-APC (clone: P44-8, 325110), CD337-APC (clone: P30-15, 325209), Tim-3 (CD366)-PE-Cy7 (clone: F38-2E2, 345014), IL-2-PE (clone: MQ1-17H12, 500307), INF-γ-APC-Cy7 (clone: B27, 506524), TCF1 (TCF7)-AF647 (clone: 7F11A10, 655203) and TCRαβ-APC (clone: IP26, 306718)
	BD Biosciences	CD4-APC-H7 (clone: RPA-T4, 560158), CD107a-APC (clone: H4A3, 560664), CD159a (NKG2A)-BV421 (clone: 131411, 747924), CD235a-APC (clone: GA-R2 (HIR2), 551336) and IL-2-APC (clone: MQ1-17H12, 561054)
	eBioscience	CD5-PE-Cy7 (clone: UCHT2, 25-0059-42) and CD43-PE (clone: eBio84-3C1, 12-0439-42)
1:10	BD Biosciences	CD45-APC (clone: HI30, 555485), CD144-FITC (clone: 55-7H1, 560411)
	Beckman Coulter	CD8β-PE (clone: 2ST8.5H7, IM2217U), Beta Mark TCR Vβ Repertoire Kit (IM3497)

Dilution	Manufacture	Anti-mouse antibody
1:40	BioLegend	CD45-PE (clone: 30-F11, 103106)

Supplementary Table 3 I The dilutions of antibodies for flow cytometry staining.

Gene	Direction	Sequence
GAPDH	Forward	GCTCTCTGCTCCTCCTGTTC
	Reverse	ACGACCAAATCCGTTGACTC
RAG1	Forward	GAGCAAGGTACCTCAGCCAG
	Reverse	AACAATGGCTGAGTTGGGAC
RAG2	Forward	GATTCCTGCTACCTCCCTCC
	Reverse	AGCGTCCTCCAAAGAGAACA

Supplementary Table 4 l The primers used for quantification of *RAG* expression.
