# Supplementary material for: The therapeutic potential of multiclonal tumoricidal T cells derived from tumor infiltrating lymphocyte-derived iPS cells
Source: Commun Biol. 2021 Jun 7;4:694. doi: 10.1038/s42003-021-02195-x (PMC8184746; doi:10.1038/s42003-021-02195-x)
Supplement: Supplementary file 3 — Description of Additional Supplementary Files [file 42003_2021_2195_MOESM3_ESM.pdf]

Description of Additional Supplementary Files

File name: Supplementary Movie 1 | *Time lapse killing image of TIL-iPS-T.*

Description: TIL-iPS-T for C-T-10 Vβ4 was co-cultured with Luc-GFP+ cancer spheroids. Propidium iodide (PI) was used for staining dead cells.

File name: Supplementary Data 1

Description: Data for figures excepting Fig. 4a, b.

File name: Supplementary Data 2

Description: Data for Fig. 4a, b.

File name: Supplementary Data 3

Description: Data for supplementary figures.
