# Supplementary material for: The therapeutic potential of multiclonal tumoricidal T cells derived from tumor infiltrating lymphocyte-derived iPS cells
Source: Commun Biol. 2021 Jun 7;4:694. doi: 10.1038/s42003-021-02195-x (PMC8184746; doi:10.1038/s42003-021-02195-x)
Supplement: Supplementary file 8 — Reporting Summary [file 42003_2021_2195_MOESM8_ESM.pdf]

Corresponding author(s): Shin Kaneko
Last updated by author(s): May 4, 2021

Reporting Summary

Nature Research wishes to improve the reproducibility of the work that we publish. This form provides structure for consistency and transparency in reporting. For further information on Nature Research policies, see our Editorial Policies and the Editorial Policy Checklist.

Statistics

For all statistical analyses, confirm that the following items are present in the figure legend, table legend, main text, or Methods section.

n/a	Confirmed	
☐	☒	The exact sample size (n) for each experimental group/condition, given as a discrete number and unit of measurement
☐	☒	A statement on whether measurements were taken from distinct samples or whether the same sample was measured repeatedly
☐	☒	The statistical test(s) used AND whether they are one- or two-sided<br>*Only common tests should be described solely by name; describe more complex techniques in the Methods section.*
☒	☐	A description of all covariates tested
☐	☒	A description of any assumptions or corrections, such as tests of normality and adjustment for multiple comparisons
☐	☒	A full description of the statistical parameters including central tendency (e.g. means) or other basic estimates (e.g. regression coefficient) AND variation (e.g. standard deviation) or associated estimates of uncertainty (e.g. confidence intervals)
☐	☒	For null hypothesis testing, the test statistic (e.g. F, t, r) with confidence intervals, effect sizes, degrees of freedom and P value noted<br>*Give P values as exact values whenever suitable.*
☒	☐	For Bayesian analysis, information on the choice of priors and Markov chain Monte Carlo settings
☒	☐	For hierarchical and complex designs, identification of the appropriate level for tests and full reporting of outcomes
☒	☐	Estimates of effect sizes (e.g. Cohen's d, Pearson's r), indicating how they were calculated

Our web collection on statistics for biologists contains articles on many of the points above.

Software and code

Policy information about availability of computer code

Data collection	BD FACSDiva 9.0 for LSRFortessa, BD FACSDiva 8.0.3 for FACSAria flow cytometer, MicroBeta2 Windows Workstation Version 2.0.0.87, MikroWin 2000 Version 4.41 for Centro LB960, Seahorse XF96 1.4.0.12, biostation, Living Image 4.0 for IVIS spectrum, Living Image 4.7.3 for IVIS Lumina, BioStation IM v2.2.1.144, BZ-X810
Data analysis	Microsoft Excel 2013 under Windows 8.1, FlowJo v10.4, FCAP Array v3.0.1, GraphPad Prism 8.4.2, Wave 2.6.0, EDIUS Neo 3.5, living Image 4.5.5, BZ-X800 Analyzer 1.1.1.8

For manuscripts utilizing custom algorithms or software that are central to the research but not yet described in published literature, software must be made available to editors and reviewers. We strongly encourage code deposition in a community repository (e.g. GitHub). See the Nature Research guidelines for submitting code & software for further information.

Data

Policy information about availability of data

All manuscripts must include a data availability statement. This statement should provide the following information, where applicable:

- Accession codes, unique identifiers, or web links for publicly available datasets
- A list of figures that have associated raw data
- A description of any restrictions on data availability

The datasets generated and analyzed during the current study are available from Supplementary Data 1-3 or from the corresponding author upon reasonable request.

Field-specific reporting

Please select the one below that is the best fit for your research. If you are not sure, read the appropriate sections before making your selection.

☒ Life sciences ☐ Behavioural & social sciences ☐ Ecological, evolutionary & environmental sciences

For a reference copy of the document with all sections, see nature.com/documents/nr-reporting-summary-flat.pdf

Life sciences study design

All studies must disclose on these points even when the disclosure is negative.

Sample size	No sample-size calculation was performed before clinical sample collection, but sample size were estimated based on the previous reports about TIL profiling.<br>3 independent cases for MMR-D (n=1) and MMR-P (n=2) were selected to verify the reproducibility of TIL-iPS-T regeneration.<br>In Supplementary Fig. 9, the number of PDSX mice was restricted due to technical difficulty of TI-CTL preparation.
Data exclusions	Two cases (C-T-1 and C-T-11) were excluded from TIL profiling cohorts because of technical failure.<br>In Fig. 6b, Vβ4 TI-CTL was not evaluated due to technical difficulty of the preparation.
Replication	In vitro assays, experiments were performed more than twice as far as primary TI-CTL were available.
Randomization	In Supplementary Fig. 9, even allocation of each groups was done based on luminescence levels and tumor volumes of the tumors.
Blinding	In Supplementary Fig. 9, an evaluator was arranged and independent of the investigator, but blinding was not possible.

Reporting for specific materials, systems and methods

We require information from authors about some types of materials, experimental systems and methods used in many studies. Here, indicate whether each material, system or method listed is relevant to your study. If you are not sure if a list item applies to your research, read the appropriate section before selecting a response.

Materials & experimental systems

n/a	Involved in the study	
☐	☒	Antibodies
☐	☒	Eukaryotic cell lines
☒	☐	Palaeontology and archaeology
☐	☒	Animals and other organisms
☐	☒	Human research participants
☐	☒	Clinical data
☒	☐	Dual use research of concern

Methods

n/a	Involved in the study	
☒	☐	ChIP-seq
☐	☒	Flow cytometry
☒	☐	MRI-based neuroimaging

Antibodies

Antibodies used

Anti-human antibodies for flow cytometry
From BioLegend: CD3-BV510 (clone: UCHT1, 300448), CD3-APC (clone: UCHT1, 300412), CD3-APC-Cy7 (clone: UCHT1, 300426), CD4-BV421 (clone: OKT4, 317434), CD7-FITC (clone: CD7-6B7, 343104), CD8-PerCP-Cy5.5 (clone: SK1, 344710), CD14-PE-Cy7 (clone: HCD14, 325618), CD16-FITC (clone: 3G8, 302006), CD27-APC (clone: O323, 302810), CD28-BV421 (clone: CD28.2, 302930), CD34-PB (clone: 581, 343512), CD45-BV421 (clone: HI30, 304031), CD45-BV510 (clone: HI30, 304036), CD45RA-BV510 (clone: HI100, 304142), CD56-APC-Cy7 (clone: HCD56, 318332), CD62L-PE-Cy7 (clone: DREG-56, 304822), CD95-PE-Cy7 (clone: DX2, 305622), CD107a-PE (clone: H4A3, 320608), 4-1BB (CD137)-PE (clone: 4B4-1, 309804), 4-1BB (CD137)-APC (clone: 4B4-1, 309810), CD158-APC (clone: HP-MA4, 339509), CD158a/h-FITC (clone: HP-MA4, 339503), CD158b-APC (clone: DX27, 312715), CD158e1-APC (clone: DX9, 312715), CD161-PE-Cy7 (clone: HP-3G10, 339918), CCR7 (CD197)-APC (clone: G043H7, 353214), LAG-3 (CD223)-FITC (clone: 11C3C65, 369308), CD226 (DNAM-1)-BV421 (clone: 11A8, 338332), PD-1 (CD279)-BV421 (clone: EH12.2H7, 329920), CD314 (NKG2D)-PE-Cy7 (clone: 1D11, 320811), CD335-FITC (clone: 29A1.4, 331921), CD336-APC (clone: P44-8, 325110), CD337-APC (clone: P30-15, 325209), Tim-3 (CD366)-PE-Cy7 (clone: F38-2E2, 345014), IL-2-PE (clone: MQ1-17H12, 500307), INF-γ-APC-Cy7 (clone: B27, 506524), TCF1 (TCF7)-AF647 (clone: 7F11A10, 655203) and TCRαβ-APC (clone: IP26, 306718).
From BD Biosciences: CD4-APC-H7 (clone: RPA-T4, 560158), CD45-APC (clone: HI30, 555485), CD107a-APC (clone: H4A3, 560664), CD144-FITC (clone: 55-7H1, 560411), CD159a (NKG2A)-BV421 (clone: 131411, 747924), CD235a-APC (clone: GA-R2 (HIR2), 551336) and IL-2-APC (clone: MQ1-17H12, 561054).
From eBioscience: CD5-PE-Cy7 (clone: UCHT2, 25-0059-42) and CD43-PE (clone: eBio84-3C1, 12-0439-42).
From Beckman Coulter: CD8β-PE (clone: 2ST8.5H7, IM2217U).

Anti-mouse antibody for flow cytometry
From BioLegend: CD45-PE (clone: 30-F11, 103106)

	Anti-human antibody for immunostaining<br>From DAKO: CD8 (clone: C8/144B, M7103)<br><br>Functional grade anti-human antibodies<br>From BioLegend: CD3 (clone: OKT3, 317315), HLA-A, B, C (clone: w6/32, 311428)
Validation	All antibodies in this manuscript are commercially available and validated by the manufacturers.

Eukaryotic cell lines

Policy information about cell lines

Cell line source(s)	TKT3V1-7 is an iPS cell line which was established by Nishimura et. al (Cell Stem Cell, 2013).<br>GPC3 16-1 is an iPS cell line which was established by co-author (Minagawa et al., Cell Stem Cell, 2018).<br>1301 were commercially obtained from ECACC.
Authentication	None of the cell lines have been authenticated.
Mycoplasma contamination	Cell lines were tested negative for Mycoplasma contamination.
Commonly misidentified lines<br>(See ICLAC register)	No commonly misidentified cell lines were used.

Animals and other organisms

Policy information about studies involving animals; ARRIVE guidelines recommended for reporting animal research

Laboratory animals	Five or six week-old female NOD-SCID IL2Rγcnull (NSG) mice were purchased from Oriental Bio (Yokohama, Japan).
Wild animals	No wild animals were involved in this study.
Field-collected samples	No field-collected samples were involved in this study.
Ethics oversight	All animal experiments and procedures were permitted by the Kyoto University Institutional Review Board.

Note that full information on the approval of the study protocol must also be provided in the manuscript.

Human research participants

Policy information about studies involving human research participants

Population characteristics	Population characteristics were described in supplementary Table. 1.
Recruitment	Colorectal cancer patients (n=16) who underwent primary tumor resections between December 2017 and November 2018 were enrolled in this study. All samples were collected irrespective of patient's background.
Ethics oversight	This study was approved by the institutional review board of the Graduate School of Medicine, Kyoto University (Approval number: G590).

Note that full information on the approval of the study protocol must also be provided in the manuscript.

Clinical data

Policy information about clinical studies

All manuscripts should comply with the ICMJE guidelines for publication of clinical research and a completed CONSORT checklist must be included with all submissions.

Clinical trial registration	This study is not a clinical trial.
Study protocol	*Note where the full trial protocol can be accessed OR if not available, explain why.*
Data collection	*Describe the settings and locales of data collection, noting the time periods of recruitment and data collection.*
Outcomes	*Describe how you pre-defined primary and secondary outcome measures and how you assessed these measures.*

Flow Cytometry

Plots

Confirm that:

☒ The axis labels state the marker and fluorochrome used (e.g. CD4-FITC).

☒ The axis scales are clearly visible. Include numbers along axes only for bottom left plot of group (a 'group' is an analysis of identical markers).

☒ All plots are contour plots with outliers or pseudocolor plots.

☒ A numerical value for number of cells or percentage (with statistics) is provided.

Methodology

Sample preparation	All stains were performed with <1x10^6 cells per 200 µL staining buffer (PBS + 2% FBS) with antibodies, 30 min on ice in dark. The dilutions of antibodies for flow cytometry staining were described in Supplementary Table 3.
Instrument	LSRFortessa (BD) or FACSAria flow cytometer (BD)
Software	FlowJo v10.4
Cell population abundance	Populations were validated for purity by a post-sort analysis with flow cytometer.
Gating strategy	All human or mice cells were first gated on FSC/SSC according to cell size and granularity, using stained human peripheral mononuclear cells (PBMCs) as a positive control and reference for cell size, granularity and staining intensity. Unstained samples were used to set up negative gates, and stained human PBMCs were used to set up positive gates. Dead cell populations were excluded using PI staining.

☒ Tick this box to confirm that a figure exemplifying the gating strategy is provided in the Supplementary Information.
